# Acute Loss of Cited2 Impairs Nanog Expression and Decreases Self-Renewal of Mouse Embryonic Stem Cells

**DOI:** 10.1002/stem.1889

**Published:** 2014-11-06

**Authors:** Kamil R Kranc, Daniel V Oliveira, Alejandro Armesilla-Diaz, Ivette Pacheco-Leyva, Ana Catarina Matias, Ana Luisa Escapa, Chithra Subramani, Helen Wheadon, Marlene Trindade, Jennifer Nichols, Keisuke Kaji, Tariq Enver, José Bragança

**Affiliations:** a MRC Centre for Regenerative Medicine, University of EdinburghEdinburgh, United Kingdom; b Departamento de Ciências Biomédicas e Medicina, Universidade do AlgarveFaro, Portugal; c Paul O'Gorman Leukaemia Research Centre, Institute of Cancer Sciences, University of Glasgow, Gartnavel General HospitalGlasgow, United Kingdom; d Wellcome Trust-Medical Research Council Cambridge Stem Cell Institute, University of CambridgeTennis Court Road, Cambridge, United Kingdom; e Stem Cell Laboratory, UCL Cancer Institute, University College LondonPaul O'Gorman Building, London, United Kingdom; f IBB-Centro de Biomedicina Molecular e Estrutural, Universidade do Algarve, Campus de GambelasFaro, Portugal

**Keywords:** Transcriptional regulation, Self-renewal, Pluripotency, Cited2, Nanog

## Abstract

Identifying novel players of the pluripotency gene regulatory network centered on Oct4, Sox2, and Nanog as well as delineating the interactions within the complex network is key to understanding self-renewal and early cell fate commitment of embryonic stem cells (ESC). While overexpression of the transcriptional regulator Cited2 sustains ESC pluripotency, its role in ESC functions remains unclear. Here, we show that Cited2 is important for proliferation, survival, and self-renewal of mouse ESC. We position Cited2 within the pluripotency gene regulatory network by defining Nanog, Tbx3, and Klf4 as its direct targets. We also demonstrate that the defects caused by Cited2 depletion are, at least in part, rescued by Nanog constitutive expression. Finally, we demonstrate that *Cited2* is required for and enhances reprogramming of mouse embryonic fibroblasts to induced pluripotent stem cells. Stem Cells *2015;33:699–712*

## Introduction

The maintenance of pluripotency of embryonic stem cells (ESC) is orchestrated by growth factors and signaling pathways that ultimately control the optimal expression of the transcription factors Oct4, Sox2, and Nanog, master regulators of pluripotency and self-renewal [1]. In mouse ESC, leukemia inhibitory factor (LIF) is crucial for pluripotency and activates in parallel the JAK/Stat3 pathway promoting the expression of Klf4, an activator of Sox2, and the PI3K/AKT pathway stimulating Tbx3 expression which subsequently activates Nanog [2,3]. The transcriptional coactivators p300 and CREB-binding protein (CBP) intervene in pluripotency and differentiation of mouse ESC, and p300 is recruited to multiple regulatory regions of the mouse ESC genome in association with Oct4, Sox2, and Nanog [4]. Cited2, a transcriptional regulator, strongly binds to p300 and CBP and is essential for mouse embryonic development [5–9] and the maintenance of fetal and adult hematopoietic stem cells [10,11]. Cited2 overexpression, like ectopic expression of Nanog, sustains self-renewal of mouse ESC in the absence of LIF [12,13]. The mechanisms regulating Cited2 expression in mouse and human ESC are still poorly understood, but Cited2 transcriptional regulatory elements are bound by transcriptional factors critical for pluripotency such as Oct4, Sox2, Nanog, FoxP1, and Zfp206 [14–16].

Here, we establish that acute loss of *Cited2* expression in mouse ESC maintained under nondifferentiating conditions results in a rapid decline of ESC self-renewal and survival. We demonstrate that Cited2 is present on the regulatory elements of *Nanog*, *Klf4*, and *Tbx3* and controls their expression in undifferentiated ESC. The constitutive expression of *Cited2* or *Nanog* in ESC rescues both self-renewal and survival defects caused by Cited2 depletion. We therefore propose that Cited2 plays an important role in mouse ESC self-renewal, proliferation, and survival, at least in part, by directly regulating transcription of *Nanog*, *Klf4*, and *Tbx3*. Finally, we demonstrate that Cited2 is necessary for the generation of induced pluripotent stem (iPS) cells from mouse embryonic fibroblasts (MEFs) and it enhances MEF reprogramming efficiency.

## Materials and Methods

### ESC and Culture Conditions

All ESC lines were cultured on gelatine-coated plates in undifferentiating medium supplemented with LIF [12]. Derivation of mouse C2^fl/fl^ ESC from Cited2^fl/fl^ blastocysts was performed as described previously [17]. C2^fl/fl^[Cre]A, C2^fl/fl^[Cre]B, and C2^fl/fl^[Cre]C ESC are three independent cell colonies obtained from the stable transfection of C2^fl/fl^ ESC with the pPyCAGIP-CreERt plasmid (encoding Cre-ERT2 [18] subcloned into pPyCAGIP). C2^fl/fl^[Cre]A, C2^fl/fl^[Cre]B, and C2^fl/fl^[Cre]C ESC were expanded and characterized for their ability to knockout *Cited2* upon treatment with 0.5 or 1 μM of 4-hydroxytamoxifen (4HT). Similarly E14TG2A[Cre] cells were obtained by stable transfection of E14TG2A with pPyCAGIP-CreERt. E14/T cells were a gift from Professor Austin Smith (University of Cambridge, U.K.) and were described elsewhere [19]. C2^fl/fl^[Cre]/CITED2 and C2^fl/fl^[Cre]/Control ESC were, respectively, obtained by transduction of C2^fl/fl^[Cre]A or C2^fl/fl^[Cre]B ESC with lentiviral particles expressing the human CITED2 peptide and the green fluorescent protein (GFP) or the control particles expressing GFP as described elsewhere [11]. C2^fl/fl^[Cre]/Control and C2^fl/fl^[Cre]/CITED2 ESC expressing GFP were isolated by fluorescence activated cell sorting using a FACSAria II Cell Sorter (BD Bioscience, Erembodegem, Belgium, http://www.bd.com/scripts/support/search.asp?city=0&country=Belgium&divisionName=0&serviceType=0&state=0). C2^fl/fl^[Cre]/Control and C2^fl/fl^[Cre]/CITED2 ESC cells treated with 0.5 or 1 μM of 4HT were converted into *Cited2* knockout cells and named C2^Δ/Δ^[Cre]/Control and C2^Δ/Δ^[Cre]/CITED2 ESC, respectively. Stable *Cited2* knockout ESC (C2^Δ/Δ^ ESC) were obtained after sorting using a FACSAria II Cell Sorter (BD Bioscience) of GFP-positive C2^fl/fl^ ESC transiently transfected with a plasmid expressing a constitutively active Cre recombinase and the GFP. Sorted GFP-positive cells were grown in culture for approximately 4 weeks before isolation by FACS based on the fluorescence emitted by the cleavage of fluorescein-di-β-d-galactopyranoside into a fluorescein by β-galactosidase expressed in *Cited2* knockout cells [20]. Single cells were individually collected in gelatine-coated 96-well plates containing ESC culture medium. Six independent colonies were then genotyped using primers listed in Supporting Information Table S1 to detect *Cited2* Cre-recombined alleles. C2^fl/fl^/Control and C2^fl/fl^/Nanog ESC were, respectively, engineered by stable transfection and puromycin resistance selection of C2^fl/fl^ ESC with the pPyCAGIP and pPyCAGIP-Nanog, gifts from Professor Austin Smith (University of Cambridge, U.K.) described elsewhere [19]. To perform the knockout, ESC-C2^fl/fl^/Control and ESC-C2^fl/fl^/Nanog cells were transiently transfected with a plasmid expressing the Cre recombinase and GFP, and sorted based on GFP expression a day after transfection using a FACSAria II Cell Sorter (BD Bioscience), and were named C2^Δ/Δ^/Control and C2^Δ/Δ^/Nanog ESC, respectively.

### Cited2 Knockdown

The KD-Cited2 plasmid was constructed by insertion of a 375 bp cDNA fragment corresponding to the amino acids 2–123 of the human CITED2 at the BamHI of the pDoubNeo vector [21]. The KD-control vector was constructed by insertion of a 369 bp fragment of the Pol region containing a splice acceptor site of the Moloney mouse leukemia virus at the BamHI of the pDoubNeo vector (referred to as KD-empty). KD-empty, KD-control, and KD-Cited2 were transfected into E14TG2A and E14/T cells using Lipofectamine 2000 (Invitrogen). These cells were maintained in culture in the presence of G418 at 400 μg/ml for the duration of the experiments to reduce the presence of untransfected cells. Plasmids used to express *Cited2* shRNA and control shRNA were previously described [11].

### Quantitative Real-Time PCR

Total RNA was isolated using the RNeasy Mini Kit (QIAGEN, Hilden, Germany, http://www.qiagen.com/) and used to synthesize complementary DNA with qScript cDNA SuperMix (Quanta BioSciences, MD, USA, http://www.quantabio.com/page/contact.php). Quantitative real-time PCR (qPCR) assays were carried out in LightCycler LC480 (Roche) or CFX96 (Bio-Rad, Birmingham, United Kingdom, http://www.bio-rad.com/?WT.srch=1&WT.mc_id=aw-corp-eu-brand&WT.knsh_id=628e3f91-bdaa-d6e8-22d6-0000734b512f) thermocyclers using SsoFast EvaGreen Supermix (Bio-Rad) or PerfeCTa SYBR Green (Quanta BioSciences) with primers listed in Supporting Information Table S1. The primer set designated Cited2#1 (Supporting Information Table S1) was used to detect both mouse endogenous *Cited2* and human exogenous *flag-CITED2* cDNA in qPCR experiments, except when otherwise stated. The primer set designated Cited2#2 is specific for detection of endogenous mouse *Cited2* expression. Expression levels were normalized to *Gapdh* or *Tbp*. Quantitative analyses were performed independently at least three times and are shown with standard error of the mean.

### Immunochemistry

Immunocytochemistry was performed using mouse monoclonal JA22 against CITED2 (AB5155, Abcam, Cambridge, United Kingdom, http://www.abcam.com/), goat anti-Nanog (AF2729, R&D Systems, Abingdon, United Kingdom, http://www.rndsystems.com/), and mouse monoclonal anti-H3triMek9 (AB8898, Abcam) antibodies as previously described [22]. Western blotting assays performed using 15–20 μg of whole cell lysates prepared from mouse ESC as previously described [22]. Mouse monoclonal JA22, goat anti-Nanog, rabbit polyclonal anti-Oct4 (AB19857, Abcam), and mouse monoclonal anti-flagM2 (F1804, Sigma, Steinheim, Germany, http://www.sigmaaldrich.com/portugal.html) antibodies were used at 1:2,000 dilution. Loading was monitored by probing the membrane with a mouse monoclonal anti-β-tubulin antibody (T5293, Sigma) used at 1:2,000 dilution.

### Chromatin Immunoprecipitation Assays

Chromatin immunoprecipitation (ChIP) experiments were performed using mouse monoclonal JA22 against CITED2 (Abcam), anti-flagM2 (Sigma), and mouse monoclonal anti-cytochrome c (Abcam), as previously described [7]. DNA enrichments were determined by qPCR using primers listed in Supporting Information Table S1.

### Transient Transfection Assays

E14TG2A cells were plated in gelatine-coated 24-well plates at 1.25 × 10^4^ cells per well and transfected in duplicate the following day using Lipofectamine 2000 (Invitrogen). The pCITED2-luc plasmid harboring the fragment −3,342 to +92 of the human CITED2 promoter has been described elsewhere [23]. The pNanog-luc and pOct4-luc plasmids harboring the mouse proximal Nanog promoter and the Oct4 promoter, respectively, were described previously [24]. The LTBX3-luc plasmid harboring the human TBX3 promoter has been previously described [25]. Cells were transfected with plasmids expressing flag-CITED2 (pPyCAGIP-flagCITED2), Nanog (pPyCAGIP-Nanog), or the control vector described elsewhere [13]. CMV-lacZ was cotransfected in all experiments, and luciferase and β-galactosidase activities measured 2 days post-transfection as previously described [22]. The ratio of luciferase to β-galactosidase was calculated to correct for variations in transfection efficiency.

### Alkaline Phosphatase and Cell Proliferation Assays

Alkaline phosphatase (AP) assays, and determination of the fraction (%) of stem cell colonies which corresponds to the number of AP positive colonies over the total number of colonies (AP-positive and -negative), were performed as previously described [12]. For cell proliferation assays, ESC were plated in gelatine-coated 12-well plates and counted at the indicated time points. Population doubling per passage and cumulative population doubling at each passage was calculated as described previously [26].

### Primary Reprogramming

Immortalized *Cited2*^Δ/Δ^ MEFs were plated at the density of 5 × 10^4^ per well in a six-well plate and transfected with the PB-TAP-IRI-attP2LMKOSimO, PB-CA-rtTA, and HyPBase plasmids [27], using XtremeGene HP reagent (Roche Applied Science, Penzberg, Germany, http://lifescience.roche.com/). To initiate reprogramming, culture medium was changed 24 hours after transfection to ESC complete medium containing LIF (1,000 U/ml), doxycycline (Dox, 1 μg/ml), vitamin C (10 μg/ml), and Alk inhibitor (500 nM). Medium was replaced every 2 days. The efficiency of the transfection was measured by analyzing the mOrange expression 2 days after the addition of Dox. Alkaline phosphatase (AP) assays were performed 15 days after the addition of Dox.

### Cited2 Overexpression in MKOS-Expressing MEFs

MEFs expressing the MKOS cassette and the *Nanog*-GFP reporter were generated previously [27]. MEFs were transduced with *Cited2*-MSCVneo retroviruses, and after 48 hours the cells were mixed with wild-type MEF in a 1:10 ratio. A total number of 1 × 10^5^ cells were plated per well in a six-well plate. The reprogramming was performed as described above, and iPS colony formation was monitored using the expression of *mOrange* and *Nanog-GFP*. AP staining and colony counts were performed 20 days after the initiation of the reprogramming.

### Statistical Analysis

Statistical significance was determined using two-tailed Student's *t* tests assuming unequal variance. *p* < 0.05 were considered statistically significant.

## Results

### Cited2 Is Required for ESC Proliferation, Survival, and Self-Renewal

**Figure 1:**
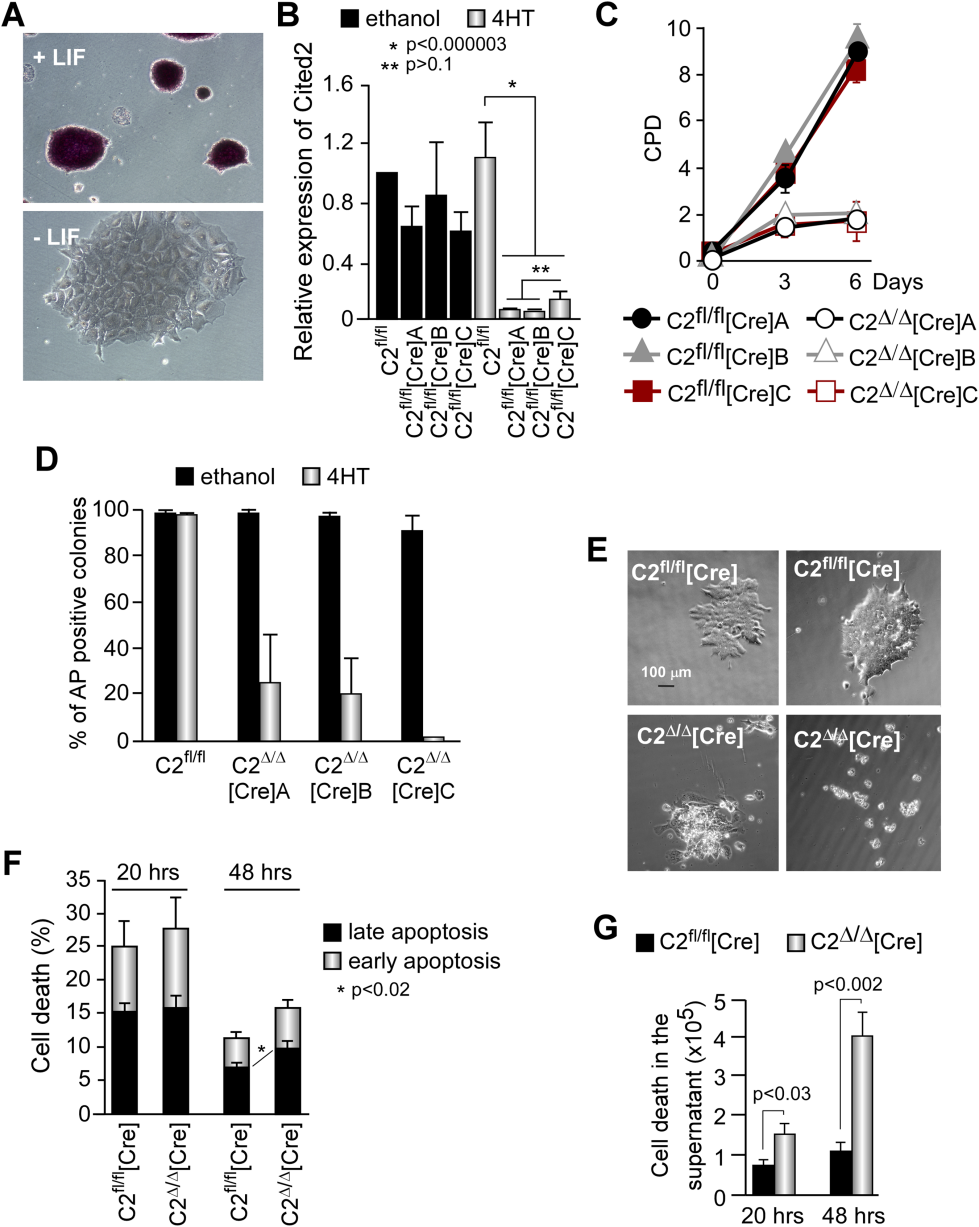
Mouse embryonic stem cells (ESC) require Cited2 for proliferation, survival, and pluripotency. (A): AP activity in C2^fl/fl^ ESC cultured on gelatine in the presence (top panel) or 4 days after removal (bottom panel) of LIF. (B): Expression of Cited2 transcripts determined by quantitative real-time PCR in C2^fl/fl^, and C2^fl/fl^ ESC stably transfected with a Cre-ERt expressing plasmid, the ESC lines C2^fl/fl^[Cre]A, C2^fl/fl^[Cre]B, and C2^fl/fl^[Cre]C, treated with 1 μM 4HT or ethanol (vehicle for 4HT) for 48 hours. Expression level is normalized for *Gapdh* and reported as relative to the expression in C2^fl/fl^ ESC treated with ethanol which is set at 1. Results are presented as the mean ± SEM of three independent biological replicates (each performed in technical duplicate). (C): Proliferation of C2^fl/fl^[Cre]A, C2^fl/fl^[Cre]B, and C2^fl/fl^[Cre]C ESC lines plated at 500 cells per well of gelatinized wells (12-well plate) in the presence of ethanol or 0.5 μM 4HT at day 0. Cells were maintained in culture with the conditions applied at day 0 until counted at the indicated time points. Results are presented as the mean ± SEM of three biological replicates, each performed in technical duplicate. (D): Percentage of AP-positive colonies over the total number of colonies at day 6 after ethanol or 4HT treatment in the presence of LIF. Results are the mean ± SEM of three independent biological replicates (each performed in technical duplicate). (E): Representative morphology of control ESC (C2^fl/fl^[Cre]) and cells 3 days after Cited2 depletion by 4HT (C2^Δ/Δ^[Cre]). (F): Percentage of C2^fl/fl^[Cre] and C2^Δ/Δ^[Cre] adherent ESC positive for Annexin V (early apopotosis) or propidium iodide (PI, late apoptosis) determined by flow sorting anlysis (FACS) 20 or 48 hours after ethanol or 4HT treatment. (G): Total number of C2^fl/fl^[Cre] and C2^Δ/Δ^[Cre] ESC in the culture supernatants stained with PI, 20 and 48 hours after treatment with ethanol or 4HT, determined by FACS. Results in (F) and (G) are shown as the mean ± SEM of three technical replicates performed with two biological replicates (i.e., C2^fl/fl^[Cre]A and C2^fl/fl^[Cre]B ESC). Abbreviations: AP, alkaline phosphatase; CPD, cumulative population doublings; LIF, leukemia inhibitory factor.

To determine the requirement for Cited2 in mouse ESC maintenance, we used a genetic loss-of-function approach. We intercrossed *Cited2*^fl/fl^ mice [20], and at the blastocyst stage, we derived ESC (hereafter called C2^fl/fl^ ESC) in which exon 2 of *Cited2* is flanked by LoxP sites. C2^fl/fl^ ESC colonies maintained in culture with LIF supplementation showed a typical ESC morphology, displayed alkaline phosphatase (AP) activity, and expressed pluripotency markers (Fig. 1A and Supporting Information Fig. S1A). Conversely, the removal of LIF or differentiation by formation of embryoid bodies caused a cellular morphology change, loss of AP activity, emergence of spontaneous beating foci, and a decrease of pluripotent markers expression with a concomitant increase of differentiation markers expression (Fig. 1A and Supporting Information Fig. S1A, S1B). A recent study showed that *Cited2*-null ESC can self-renew, but are affected in their differentiation capacities [28]. To document such cells, we transiently transfected C2^fl/fl^ ESC with a Cre and green fluorescent protein (GFP) coexpressing plasmid, sorted highly GFP-positive ESC to enhance the chances of deleting both *Cited2* alleles, and allowed the cells to grow in undifferentiating conditions for more than a month. Although, most ESC showed an impairment of growth, self-renewal and/or survival, within the first 3 days following *Cited2* deletion, a few (i.e., ∼3%) able to self-renew were genotyped as *Cited2*-null ESC, and expressed normal levels of *Nanog*, *Oct4*, *Sox2*, *Rex1*, *Klf4*, *Tbx3*, and *c-Myc* (Supporting Information Fig. S2). Therefore, as previously reported, a minority of C2^Δ/Δ^ ESC adapted to the loss of *Cited2* and managed to survive, but for the majority of the ESC, the early response to acute loss of *Cited2* expression was a substantial reduction in their proliferation suggesting that Cited2 plays a pivotal role in ESC maintenance and survival.

To further define the role of Cited2 in mouse ESC, we generated C2^fl/fl^ ESC that constitutively express tamoxifen-inducible Cre (Cre-ERt) by stable transfection and isolated three independent clones, named C2^fl/fl^[Cre]A, C2^fl/fl^[Cre]B, and C2^fl/fl^[Cre]C. Since C2^fl/fl^[Cre]A, C2^fl/fl^[Cre]B, and C2^fl/fl^[Cre]C presented indistinguishable behavior, for simplicity, they will be referred to as C2^fl/fl^[Cre] ESC. The activation of Cre-ERt by 4-hydroxytamoxifen (4HT) treatment significantly reduced endogenous *Cited2* expression within 48 hours in C2^fl/fl^[Cre] ESC while *Cited2* expression in C2^fl/fl^ ESC was maintained at levels similar to those of ethanol (vehicle) treated cells (Fig. 1B). Surprisingly, C2^Δ/Δ^[Cre] ESC also showed a concomitant decrease in *Nanog* expression while the expression of other pluripotency markers was unaffected compared to ethanol treated cells (Supporting Information Fig. S1C). In addition, C2^Δ/Δ^[Cre] ESC displayed impaired self-renewal capacity (Fig. 1C, 1D). Similarly, GFP-positive C2^fl/fl^ ESC transiently transfected with the Cre and GFP coexpressing plasmid showed impaired growth, while 4HT or ethanol treatment of E14TG2A ESC stably expressing Cre-ERt had no effect on their growth ruling out a major nonspecific effect of the activated Cre-ERt in our experimental conditions (Supporting Information Fig. S1D, S1E). Moreover, morphological colony alterations and a noticeable amount of cells detached from the plate were observed in C2^Δ/Δ^[Cre] cultures (Fig. 1E), suggesting spontaneous differentiation, increased cell death, and/or decline of cell adhesion. Staining of the adherent cells with Annexin V and propidium iodide (PI) revealed statistically significant difference in cell viability between C2^Δ/Δ^[Cre] and C2^fl/fl^[Cre] ESC cultures (Fig. 1F). Conversely, cells floating in the culture medium, 20 and 48 hours after addition of 4HT, were stained by PI indicating their late apoptotic state and were two- to fourfold more abundant in *Cited2*-knockout than control cultures (Fig. 1G). Therefore, the decrease in cell proliferation and diminished total number of Cited2-knockout cells in comparison to control cells, occurring as early as 48 hours after inducing *Cited2* deletion, results from enhanced differentiation and/or increased death of cells lacking *Cited2* expression.

**Figure 2:**
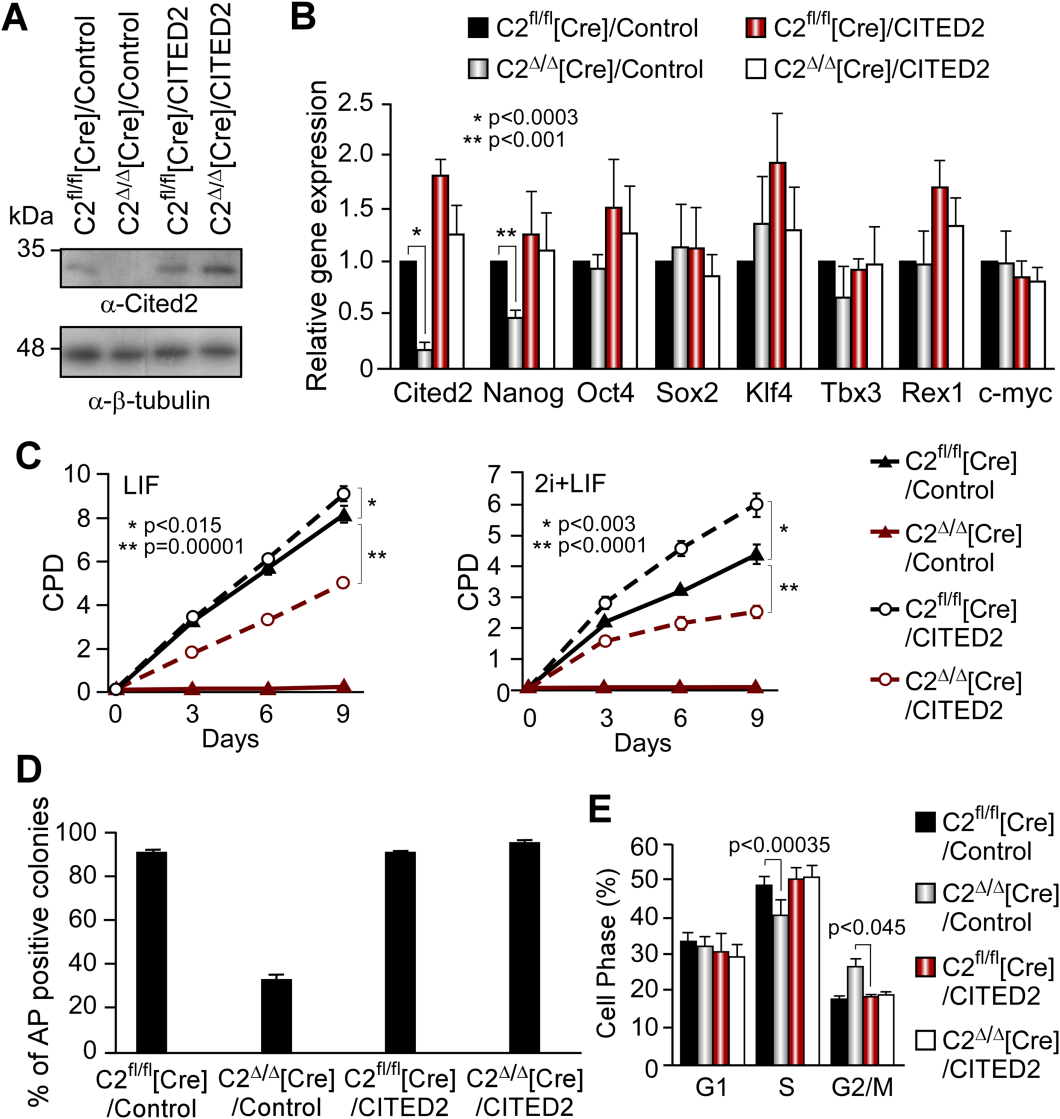
*Cited2* knockout defects in embryonic stem cells (ESC) are rescued by human CITED2 expression. (A): C2^fl/fl^[Cre]A and C2^fl/fl^[Cre]B ESC were transduced with a lentiviral vector constitutively expressing the human CITED2 or a control vector to generate C2^fl/fl^[Cre]/CITED2 and C2^fl/fl^[Cre]/Control, respectively. Cited2 protein levels were detected by Western blotting in extracts from C2^fl/fl^[Cre]/Control, C2^fl/fl^[Cre]/CITED2, C2^Δ/Δ^[Cre]/Control, and C2^Δ/Δ^[Cre]/CITED2 ESC 48 hours after incubation with ethanol or 4HT. Loading in each lane was monitored by detection of β-tubulin. (B): Relative expression of *Cited2* and pluripotency markers in cells described in (A). Gene expression in C2^fl/fl^[Cre]/Control ESC was set to 1. Results are presented as the mean ± SEM of technical triplicates performed using two independent biological replicates (i.e., C2^fl/fl^[Cre]A and C2^fl/fl^[Cre]B ESC). (C): CPD of cells as described in A (LIF) or further supplemented with 1 μM PD0325901 and 3 μM CHIR99021 (LIF+2i), after treatment with ethanol or 4HT for 24 hours. Cells were plated at 10,000 cells per gelatinized well of a six-well plate at day 0. Results are presented as the mean ± SEM of three independent experiments performed with C2^fl/fl^[Cre]B ESC. (D): Percentage of AP-positive colonies over the total number of colonies in C2^fl/fl^[Cre]/Control, C2^Δ/Δ^[Cre]/Control, C2^fl/fl^[Cre]/CITED2, and C2^Δ/Δ^[Cre]/CITED2 ESC cultures 6 days after ethanol or 4HT treatment. Results are presented as the mean ± SEM of technical triplicates performed in two independent biological replicates. (E): FACS analysis of the indicated ESC cell cycle profile by propidium iodide staining. Data are the mean ± SEM of three independent experiments performed with C2^fl/fl^[Cre]/Control, C2^Δ/Δ^[Cre]/Control, C2^fl/fl^[Cre]/CITED2, and C2^Δ/Δ^[Cre]/CITED2 ESC. Abbreviations: AP, alkaline phosphatase; CPD, cumulative population doublings; LIF, leukemia inhibitory factor.

To confirm that the defects we observed in C2^Δ/Δ^ [Cre] ESC were specific to *Cited2* deletion, we transduced C2^fl/fl^[Cre]A and C2^fl/fl^[Cre]B ESC lines with a lentiviral vector constitutively expressing GFP and the human CITED2 (with ∼95% homology to the mouse protein) which rescues developmental defects resulting from Cited2 deficiency [13]. For simplicity, these cells will be referred to as C2^fl/fl^[Cre]/CITED2 ESC. We also developed C2^fl/fl^[Cre]/Control ESC expressing GFP without exogenous CITED2. Since the data obtained using C2^fl/fl^[Cre]A and C2^fl/fl^[Cre]B cell lines were very consistent, they are presented as combined in this section. The activation of Cre-ERt by 4HT treatment significantly reduced endogenous *Cited2* expression in C2^Δ/Δ^[Cre]/Control ESC, while overall CITED2 expression in C2^fl/fl^[Cre]/CITED2 and C2^Δ/Δ^[Cre]/CITED2 ESC was maintained at levels similar to those of ethanol treated C2^fl/fl^[Cre]/Control ESC (Fig. 2A, 2B). Similarly to C2^Δ/Δ^[Cre] cells, C2^Δ/Δ^[Cre]/Control ESC also showed a decreased *Nanog* expression while the expression of other pluripotency markers was unaffected compared to C2^fl/fl^[Cre]/Control ESC (Fig. 2B). Like C2^Δ/Δ^[Cre] cells, C2^Δ/Δ^[Cre]/Control ESC displayed impaired growth suggesting that lentiviral integration events used to originate C2^fl/fl^[Cre]/Control did not alter C2^fl/fl^[Cre] ESC properties (Fig. 2C). Similar effects were obtained with medium supplemented with LIF, or LIF and inhibitors of MEK and GSK3 (2i) which are essential for the maintenance of ground state pluripotency of ESC [29], and with fetal bovine serum from different providers (Fig. 2C and Supporting Information Fig. S1H), suggesting that Cited2 is required for the survival of ESC in stringent naive conditions and its effects did not vary with serum batches. In addition, C2^Δ/Δ^[Cre]/Control ESC displayed reduced AP activity (Fig. 2D and Supporting Information Fig. S1G). Importantly, both impaired growth and AP activity defects were rescued by ectopic expression of CITED2 in C2^Δ/Δ^[Cre]/CITED2 ESC, indicating that they are specific to *Cited2* deletion. However, the constitutive expression of CITED2 did not completely rescue defective proliferation rates of C2^Δ/Δ^[Cre] ESC to the level of control ESC expressing endogenous Cited2. This suggests that ESC optimal proliferation requires *Cited2* expression driven by its endogenous promoter.

The cell-cycle analyses revealed that 48 hours after 4HT treatment C2^Δ/Δ^[Cre]/Control ESC displayed a increase in G2/M phase and a concomitant decrease in S phase compared to C2^Δ/Δ^[Cre]/CITED2 ESC (Fig. 2E). Therefore, the decrease in cell proliferation and diminished total cell number upon *Cited2* deletion may result from G2/M cell cycle arrest, enhanced differentiation, and/or increased death of cells lacking Cited2 expression. Overall, we unequivocally demonstrated that the majority of Cited2-knockout ESC are substantially compromised in their proliferation, self-renewal, and survival within the first 2–3 days of *Cited2* deletion.

### Spontaneous Differentiation of Cited2-Knockdown ESC

**Figure 3:**
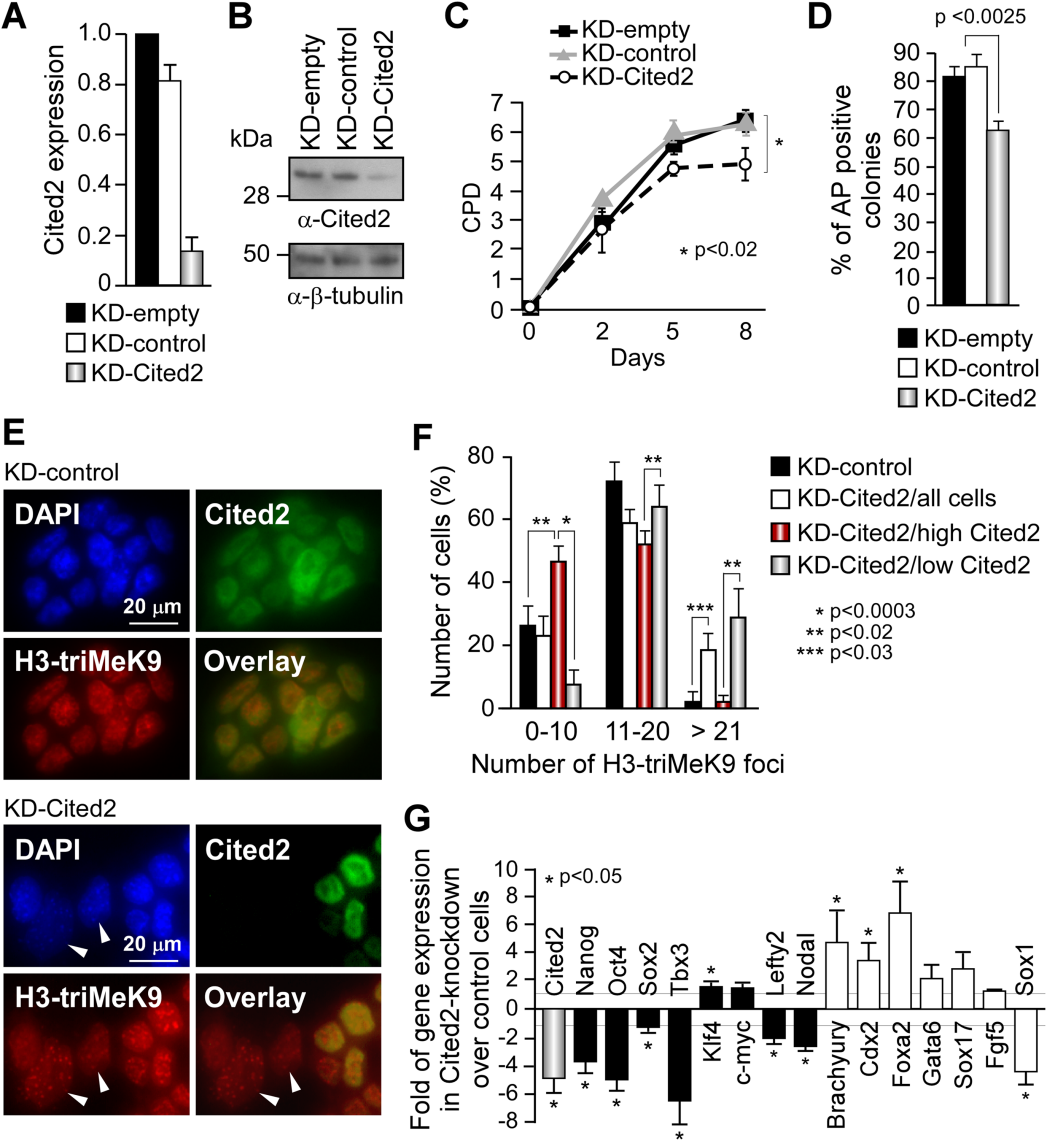
*Cited2* knockdown results in spontaneous differentiation in embryonic stem cells. (A): Relative *Cited2* gene expression in E14TG2A cells detected by quantitative real-time PCR (qPCR), 6 days post-transfection of a vector expressing a double stranded RNA targeting Cited2 (KD-Cited2), a nontargeting double stranded RNA (KD-control), and the empty (KD-empty) vectors. Cited2 expression in E14TG2A cells with the empty vector is set to 1. Results are presented as the mean ± SEM of three independent experiments performed in technical triplicates. (B): Cited2 protein levels detected in E14TG2A extracts with anti-Cited2 antibody by Western blotting. Equal loading in each lane was monitored by β-tubulin detection. (C): Proliferation of E14TG2A cells transfected with KD-empty, KD-control, or KD-Cited2 vectors. Cells were plated at 500 cells per gelatinized wells (12-well plate) the day after transfection. Cells were maintained in culture under the conditions applied at day 0 until counted at the indicated time points. Results are presented as the mean ± SEM of three independent experiments, each performed in technical duplicate. (D): Percentage of AP-positive colonies over the total number of colonies, 5 days post-transfection of E14TG2A cells with KD-empty, KD-control, or KD-Cited2 vectors. Results are presented as the mean ± SEM of three biological experiments. (E): KD-control or KD-Cited2 transfected E14TG2A cells coimmunostained with anti-Cited2 (green), anti-H3-triMeK9 (red) antibodies, and DAPI (blue). Arrow heads indicate cells with undetectable Cited2 protein, with enlarged nuclei and increased number of H3-triMeK9 foci. (F): Distribution of H3-triMeK9 foci number in KD-control or KD-Cited2 transfected cells described in (E). The results are presented as cluster of cells displaying up to 10 (0–10), between 11 and 20 (11–20), and more than 21 H3-triMeK9 foci in KD-control (black bars) and all KD-Cited2 (white bars) transfected cells. The number of H3-triMeK9 in KD-Cited2 transfected cells expressing high levels of Cited2 (red bars) and low levels or no detected Cited2 (gray bars) is also presented. (G): Transcript levels of pluripotency (black bars), mesoderm (Brachyury, Cdx2), endoderm (Foxa2, Gata6, Sox17), ectoderm (Fgf5, Sox1) markers, and Cited2 (gray bar) detected by qPCR using the primer set Cited2#2 (Supporting Information Table S1) in E14TG2A cells 6 days post-transfection of KD-empty or KD-Cited2. Gene expression presented as fold of expression relative to KD-empty treated cells. Data are the mean ± SEM of three independent experiments, each performed in technical triplicate. Abbreviation: AP, alkaline phosphatase.

To corroborate our results obtained in C2^Δ/Δ^[Cre] ESC in different ESC line, we knocked down the expression of *Cited2* in a previously established and extensively studied feeder-independent E14TG2A ESC line [30,31]. To silence *Cited2* expression in E14TG2A ESC, we used a plasmid expressing a double stranded RNA corresponding to ∼375 bp of the 5′ of the human CITED2 open reading frame (KD-Cited2). ESC were also transfected with the empty vector (KD-empty) or a vector expressing a ∼369 bp fragment (KD-control) of a nontargeting double stranded RNA. In contrast to C2^Δ/Δ^[Cre] ESC which were compromised in their survival, E14TG2A ESC transfected with KD-Cited2 did not present apparent cell death, although Cited2 expression was reduced to 15%–20% of its normal expression 6 days after transfection (Fig. 3A, 3B). Conversely, Cited2-knockdown in E14TG2A ESC caused a reduction of proliferation and a marked reduction of the number of AP-positive colonies in comparison to cells transfected with the control KD-empty or KD-control vectors (Fig. 3C, 3D and Supporting Information Fig. S1F). The decrease of AP activity and the increase of histone H3K9 trimethylated nuclear foci (Fig. 3E, 3F), a mark of ESC differentiation [32], suggested a spontaneous differentiation of Cited2-knockdown cells maintained in conditions sustaining an undifferentiated state. The differentiation was further supported by the concomitant expression of differentiation markers, such as *Brachyury*, *Cdx2* (mesoderm), and *Foxa2* (endoderm), while the expression of *Gata6* and *Sox17*, extraembryonic endodermal markers, and *Fgf5*, primitive ectodermal marker, was unaffected whereas *Sox1* (definitive ectoderm) expression was decreased in Cited2-knockdown cells (Fig. 3G). Six days after Cited2-knockdown in E14TG2A, the decrease of pluripotency markers expression such as *Oct4*, *Nanog*, *Tbx3*, *Lefty2*, and *Nodal* was also observed, confirming further the differentiation of Cited2-knockdown cells (Fig. 3G). However, the decrease of pluripotency gene expression in Cited2-knockdown cells might result either from a direct effect of Cited2-depletion or is a consequence of the spontaneous differentiation process initiated by Cited2-knockdown. Collectively, these observations demonstrated that E14TG2A ESC with reduced levels of Cited2 expression were compromised in their ability to remain undifferentiated in conditions supporting ESC self-renewal.

### Cited2 Regulates Nanog Expression in Mouse ESC

**Figure 4:**
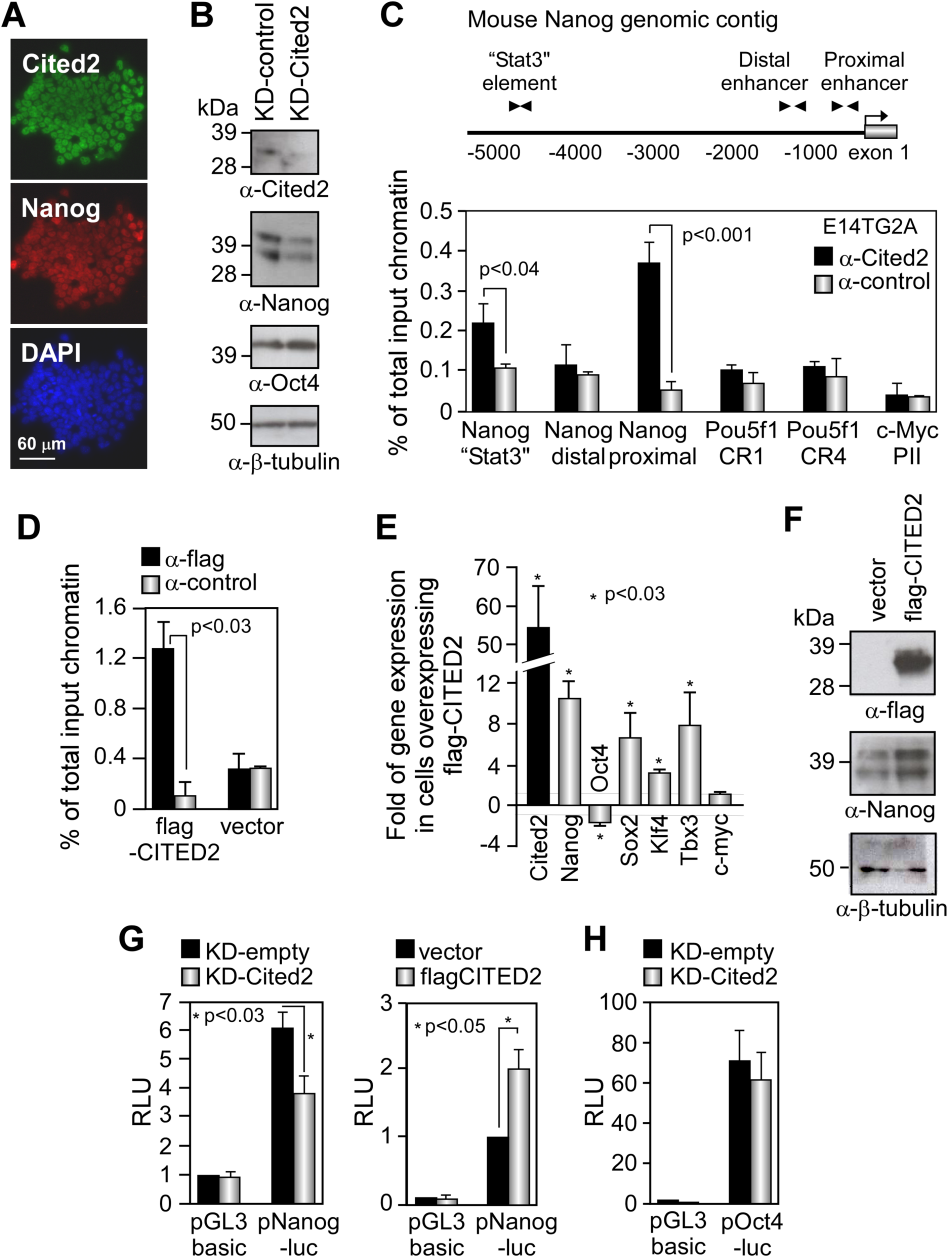
Cited2 controls *Nanog* expression. (A): Immunocytochemistry using anti-Cited2 (green), anti-Nanog (red) antibodies, and DAPI (blue) in E14TG2A. (B): Cited2, Nanog, and Oct4 protein levels detected in E14TG2A extracts prepared 48 hours post-transfection with KD-control or KD-Cited2. Loading in each lane was monitored by detection of β-tubulin. (C): Top: diagram of the mouse Nanog genomic contig showing the transcriptional start site (arrow), exon 1 (gray box), and the positions of PCR primers (arrow heads) used in chromatin immunoprecipitation (ChIP) assays. Bottom: enrichment analyzed by quantitative real-time PCR (qPCR) of the “Stat3” element, the distal enhancer and the proximal promoter of Nanog, as well as the CR1 proximal promoter and the CR4 distal enhancer of Oct4, and c-Myc proximal promoter in ChIP assays using anti-Cited2 and control anti-flag antibodies. Results are presented as the mean ± SEM of three independent experiments. (D): Enrichment of Nanog proximal promoter from E14/T cells expressing flag-CITED2 or control vector in ChIP assays using anti-flag or control antibodies. Results are presented as the mean ± SEM of three biological experiments. (E): Endogenous Nanog, Oct4, Sox2, Klf4, Tbx3, c-Myc, and ectopic CITED2 transcript levels detected by qPCR in E14/T cells transfected with pPyCAGIP or pPyCAGIP-flagCITED2. Expression is presented as fold relative to pPyCAGIP. Results are shown as the mean ± SEM of three independent experiments. (F): Ectopic flag-CITED2 and endogenous Nanog protein levels in E14/T embryonic stem cells (ESC) transfected with pPyCAGIP or pPyCAGIP-flagCITED2 detected by Western blotting. Loading in each lane was monitored by detection of β-tubulin. (G): Left panel: pNanog-luc or pGL3basic activity in E14TG2A cells cotransfected with KD-Cited2 (gray bars) or KD-empty (black bars). RLU are presented relative to RLU of pGL3basic transfected with the control vector KD-empty set at 1. Right panel: pNanog-luc or pGL3basic activity in E14TG2A cells cotransfected with pPyCAGIP-flagCITED2 (gray bars) or pPyCAGIP (black bars). Results are presented as the mean ± SEM of three biological experiments performed in duplicate. (H): pOct4-luc or pGL3basic activity in E14TG2A ESC cotransfected as in (G). Results are presented as the mean ± SEM of three independent experiments (each performed in technical duplicate). Abbreviation: RLU, relative luminescence units.

Nanog and Cited2 were detected in ESC nuclei (Fig. 4A), and their expression was concomitantly decreased in E14TG2A, C2^Δ/Δ^[Cre], and C2^Δ/Δ^[Cre]/Control ESC 2 days after Cited2 depletion, while *Oct4* expression was unaffected (Figs. 2B, 4B and Supporting Information Fig. S1C). To determine whether Cited2 directly controls the expression of Nanog, we tested the recruitment of Cited2 to the Nanog promoter elements in undifferentiated ESC by ChIP assay. A significant enrichment of the Nanog proximal promoter and the “Stat3-binding” element was detected from extracts immunoprecipitated with the anti-Cited2 antibody (Fig. 4C), revealing the presence of endogenous Cited2 at these regulatory elements. No enrichment of c-Myc promoter was detected with anti-Cited2 antibody suggesting that Cited2 is specifically recruited to the Nanog elements. To investigate the effect of Cited2 overexpression in Nanog expression, we used E14/T ESC derived from E14TG2A cells which express the polyoma large T protein and permit the stable episomal expression of plasmids containing the origin of DNA replication of the polyoma virus, such as pPyCAGIP-derived plasmids [19]. In E14/T ESC forced to express high levels of flag-CITED2 and displaying enhanced Nanog expression (Fig. 4E, 4F), we observed an augmented recruitment of flag-CITED2 to the proximal promoter of Nanog when compared with endogenous Cited2 binding, suggesting a dose-dependent enrolment of Cited2 to the regulatory elements of *Nanog* (Fig. 4D). In reporter assays, we showed that the Nanog proximal promoter activity was reduced by Cited2 depletion, while significantly enhanced by expression of flag-CITED2 (Fig. 4G). In contrast, Cited2 depletion or overexpression had little or no effect on Oct4 promoter transcriptional activity, which is in line with the lack of enrichment of the Oct4 regulatory elements in ChIP assays with anti-Cited2 antibody (Fig. 4C, 4H), indicating further that *Nanog* is a specific Cited2 target gene. Overall, our results indicated that in undifferentiated mouse ESC, Cited2 directly stimulates *Nanog* expression by acting positively on Nanog promoter, and suggest that the decrease in *Oct4* expression occurring in ESC after prolonged Cited2 depletion (Fig. 3G) is an indirect consequence of *Nanog* downregulation and/or a result of spontaneous differentiation.

### Cited2 Modulates the Expression of Tbx3 and Klf4 in Mouse ESC

**Figure 5:**
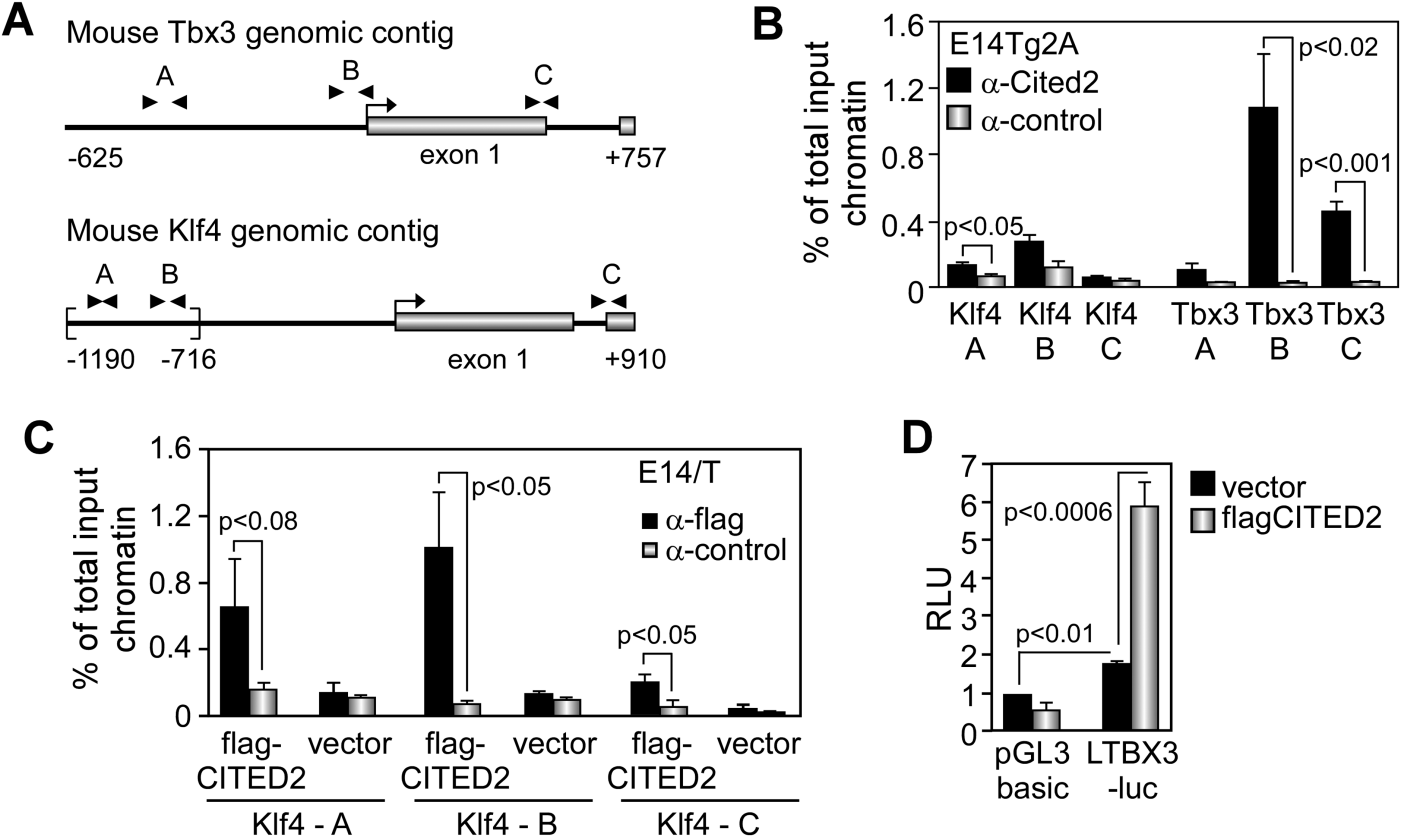
Cited2 is present at the Klf4 and Tbx3 promoters in embryonic stem cells (ESC). (A): Diagram of the mouse *Klf4* and *Tbx3* genomic contigs showing the transcriptional start site (arrow), exon 1 (gray box), and the positions of PCR primers (arrow heads) used in chromatin immunoprecipitation (ChIP) assays. The region of mouse Klf4 promoter responsive to Cited2 overexpression in epithelial cells determined elsewhere is indicated by brackets. (B): Enrichment of the promoter region of *Klf4* (Klf4 A and B), the junction of intron 1 and exon 2 of Klf4 (Klf4 C), the distal (Tbx3 A), proximal promoter (Tbx3 B), and the junction of exon 1 and intron 1 of Tbx3 (Tbx3 C) in ChIP assays with anti-Cited2 compared to control antibodies. (C): ChIP assays using anti-flag or control antibodies as described above with Klf4 primers in E14/T expressing flag-CITED2 or control vector. (D): LTBX3-luc or pGL3basic activity in E14TG2A ESC cotransfected as in Figure 4G. Data are the mean ± SEM of three independent experiments (each performed in technical duplicate). Results in (B) and (C) are presented as the mean ± SEM of three independent biological experiments. Abbreviation: RLU, relative luminescence units.

**Figure 6:**
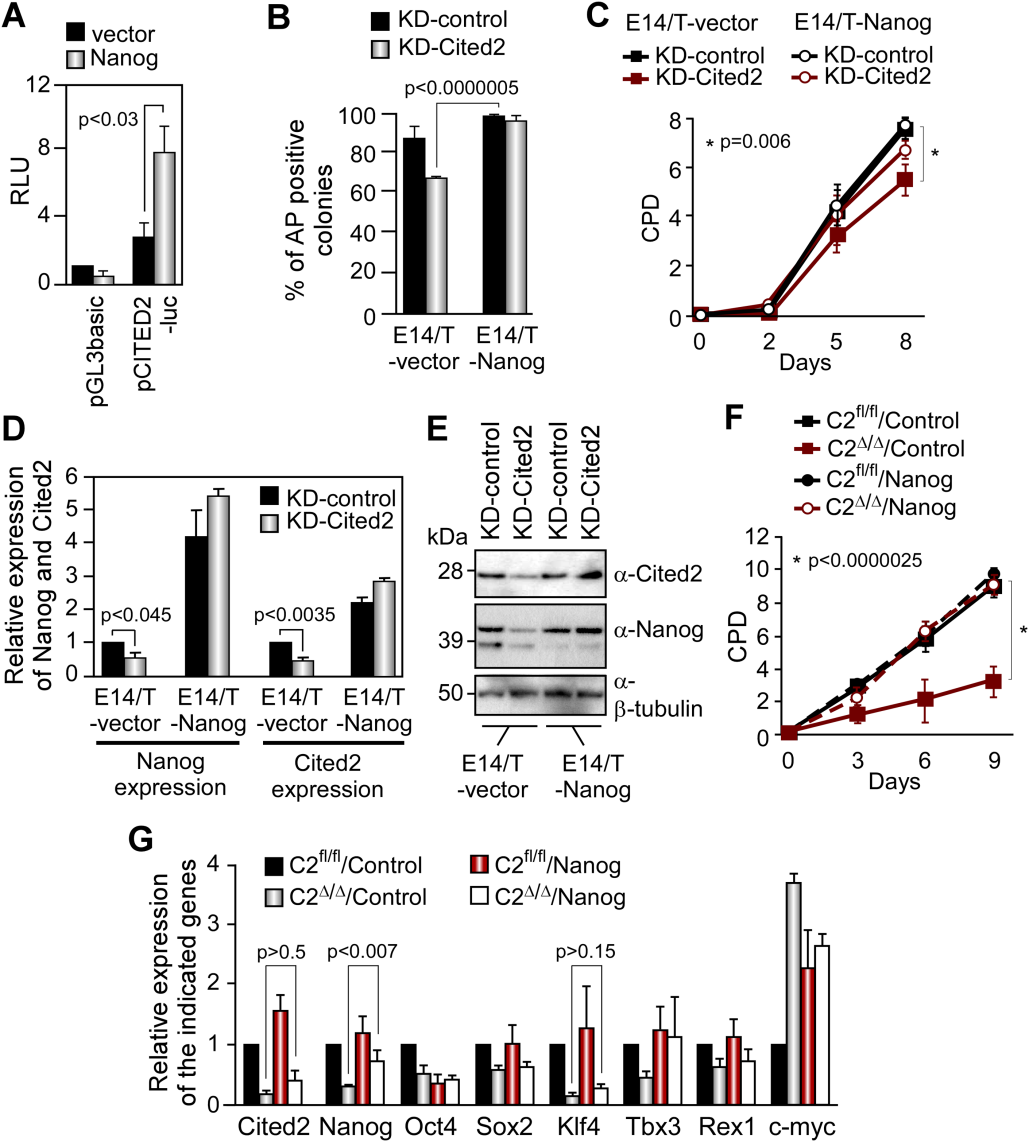
Nanog overexpression rescues defects caused by knockdown of *Cited2*. (A): pCITED2-luc or pGL3basic activity in E14TG2A embryonic stem cells (ESC) cotransfected with pPyCAGIP (black bars) or pPyCAGIP-Nanog (gray bars). RLU are presented relative to RLU of pGL3basic transfected with the control vector pPyCAGIP set at 1. Results are shown as the mean ± SEM of three independent experiments. (B): Percentage of AP-positive colonies over the total number of colonies in E14/T-vector and E14/T-Nanog cells transfected with KD-control or KD-Cited2 vectors. Results are presented as the mean ± SEM of three biological experiments. (C): Proliferation of E14/T-vector and E14/T-Nanog treated as in (B). Results are presented as the mean ± SEM of three independent experiments. (D): Nanog and Cited2 transcript levels detected by quantitative real-time PCR (qPCR) in E14/T-vector or E14/T-Nanog cells transfected for 48 hours KD-control or KD-Cited2. Gene expression is presented relative to expression in E14/T-vector (which is set at 1). Results are presented as the mean ± SEM of three independent experiments. (E): Cited2 and Nanog protein levels in E14/T-vector or E14/T-Nanog cell extracts. Equal loading in each lane was monitored by β-tubulin detection. (F): Proliferation of C2^fl/fl^/Control or C2^fl/fl^/Nanog ESC as well as C2^Δ/Δ^/Control and C2^Δ/Δ^/Nanog ESC isolated 48 hours post-transfection of a Cre and green fluorescent protein (GFP) expressing plasmid. Sorted GFP^+^ cells were plated at 10,000 cells per gelatinized wells (six-well plate) at day 0. Results are presented as the mean ± SEM of three independent experiments. (G): Cited2, Nanog, Oct4, Sox2, Rex1, Tbx3, Klf4, and c-Myc transcript levels detected by qPCR from C2^fl/fl^/Control, C2^fl/fl^/Nanog, C2^Δ/Δ^/Control, and C2^Δ/Δ^/Nanog GFP-positive cells purified by FACS, 4 days post-transfection of a Cre and GFP coexpressing plasmid. Data are the mean ± SEM of three independent experiments. Abbreviations: AP, alkaline phosphatase; CPD, cumulative population doublings; RLU, relative luminescence units.

CITED2 overexpression in E14/T ESC also increased Sox2, Klf4, and Tbx3 expression (Fig. 4E), whereas Cited2 depletion resulted in a significant decrease of *Klf4* and *Tbx3* expression (Figs. 3G, 6G). Using ChIP assays, we showed that endogenous Cited2 was present at the distal region of the Klf4 promoter (Fig. 5), which is homologous to the previously identified CITED2-responsive region of the human KLF4 promoter [33]. The recruitment of flag-CITED2 at the *Klf4* locus correlated with the increase of the endogenous Klf4 expression in E14/T cells overexpressing flag-CITED2. The presence of Cited2 was also detected at the proximal promoter and the 3′ end of the first exon of the *Tbx3* locus, and flag-CITED2 overexpression stimulated the transcriptional activity of the human *TBX3* promoter in reporter assays (Fig. 5B, 5D). Therefore, *Tbx3* and *Klf4* are direct downstream targets of Cited2 in ESC, suggesting that Cited2 supports LIF-activated PI3K/AKT/Tbx3/Nanog and Jak/Stat3/Klf4/Sox2 pathways that are critical for mouse ESC maintenance [2,3].

### Ectopic Nanog Expression Bypasses Defects Caused by Cited2 Depletion

Previous studies indicated the presence of NANOG at the endogenous CITED2 promoter in human ESC [14]. Concordantly, we showed that Nanog stimulated the human *CITED2* promoter activity (Fig. 6A). These observations, together with the data showing that Cited2 also acts upstream of Nanog, suggest a complex reciprocal interaction between Nanog and Cited2. To explore the epistatic relationship between *Cited2* and *Nanog*, we asked whether forced expression of Nanog can restore normal ESC functions of Cited2-knockdown cells. Indeed, Cited2-knockdown E14/T ESC constitutively expressing Nanog generated significantly higher numbers of AP-positive colonies and had increased growth rate compared to Cited2-knockdown E14/T-vector cells (Fig. 6B–6E). However, in line with the reporter assays, Nanog overexpression increased Cited2 expression in E14/T-Nanog cells (Fig. 6D). Moreover, the ectopic expression of Nanog in these conditions is higher than endogenous control levels even in Cited2-knockdown ESC. Thus, to verify whether Nanog can autonomously rescue the defects in absence of Cited2, we engineered C2^fl/fl^ ESC stably expressing Nanog (C2^fl/fl^/Nanog ESC) at levels comparable to those of control cells (C2^fl/fl^/Control ESC), and deleted *Cited2* by transfecting a Cre-expressing plasmid, which per se did not perturb cellular growth of ESC (Supporting Information Fig. S1D). *Cited2* deletion impaired the proliferation of control cells, C2^Δ/Δ^/Control ESC, but not Cited2-deficient C2^Δ/Δ^/Nanog ESC (Fig. 6F). As expected, *Cited2* deletion in C2^Δ/Δ^/Control ESC caused a significant decrease of Cited2 and Nanog expression compared to the control C2^fl/fl^/Control cells 4 days after the transfection of the Cre-expressing plasmid (Fig. 6G). A concomitant decrease of Klf4 expression was observed in C2^Δ/Δ^/Control ESC, 4 days after the transfection of the Cre-expressing plasmid, that was not observed in C2^Δ/Δ^ [Cre]/Control ESC 2 days after *Cited2*-knockout (Fig. 2B). Ectopic Nanog expression in *Cited2*-null ESC restored Nanog expression levels and increased *c-Myc* expression. Conversely, *Klf4* expression remained low in Nanog-rescued cells, which is in line with previous reports that showed that Klf4 is not a direct target of Nanog [34], and suggesting that Cited2 activates *Klf4* expression in a Nanog-independent manner. Therefore, our results showed that Cited2 and Nanog may be part of a feed-forward transcriptional loop required for ESC self-renewal, and the defects caused by Cited2 depletion are, at least in part, due to Nanog downregulation.

### Cited2 Is Required for and Enhances Reprogramming of MEFs into iPS Cells

**Figure 7:**
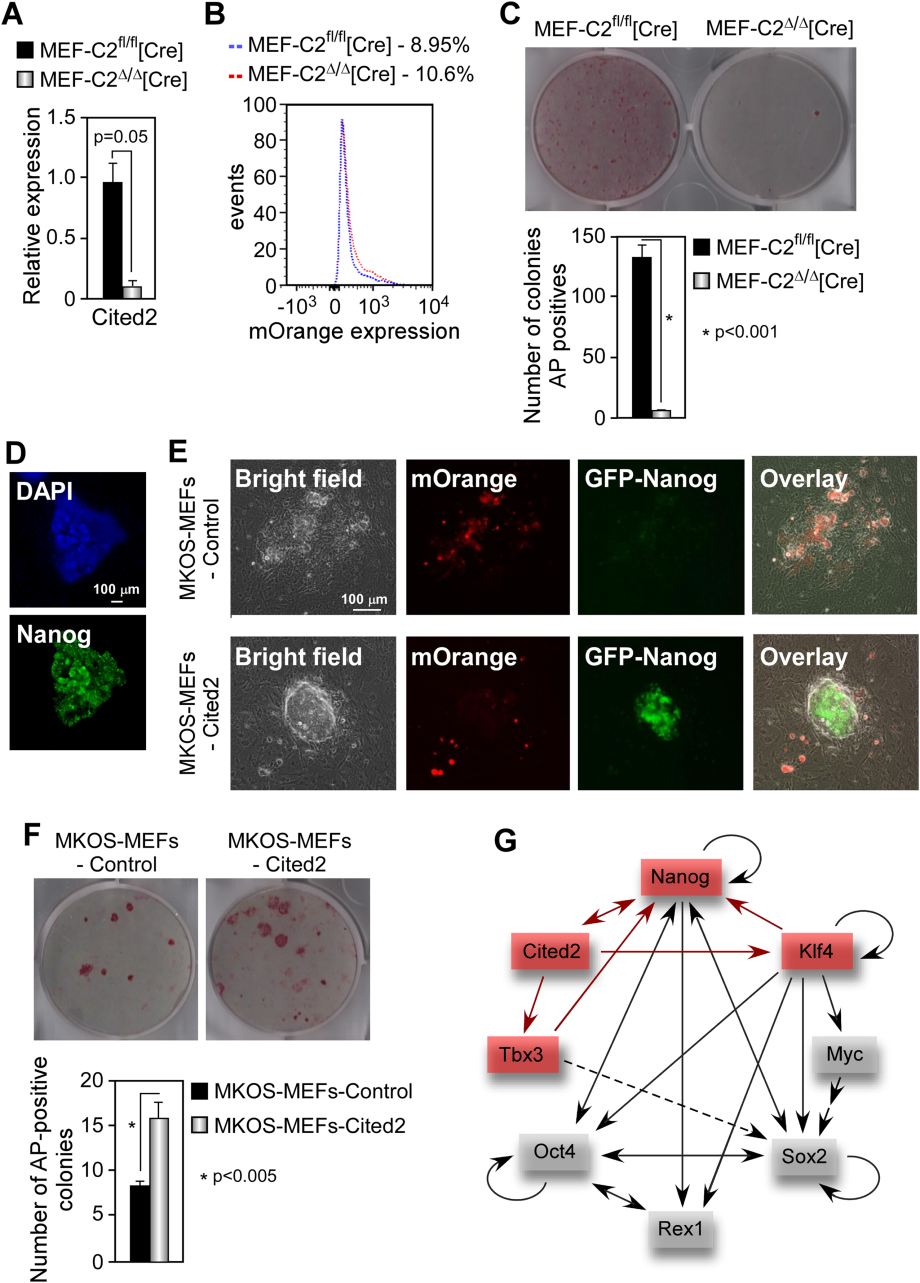
*Cited2* is essential for the generation of induced pluripotent stem cells and enhances the efficiency of reprogramming. (A): Relative expression of *Cited2* mRNA in control (MEF-C2^fl/fl^[Cre]) and *Cited2*^Δ/Δ^ (MEF-C2^Δ/Δ^[Cre]) MEFs measured by quantitative real-time PCR using the Cited2#3 primer set and normalized to *Tbp* expression (Supporting Information Table S1). (B): mOrange expression in control and *Cited2*^Δ/Δ^ MEFs 2 days after transfection of PB transposon harboring *MKOS-IRES-mOrange* cassette, a constitutively active *CAG-rtTA* transactivator construct and a transposase expression vector. (C): AP staining following 15 days in culture in the presence of doxycycline. (D): Nanog expression in primary colonies 15 days after doxycycline treatment. (E): MEFs harboring a doxycycline-inducible MKOS-IRES-mOrange cassette and *Nanog-eGFP* reporter (MKOS-MEFs) were transduced with MSCV retroviruses expressing *Cited2* or empty control retroviruses and treated with doxycycline. MKOS-MEFs transduced with *Cited2* and control retroviruses are referred to as MKOS-MEFs-Cited2 and MKOS-MEFs-Control, respectively. (F): AP staining in MKOS-MEFs-Cited2 or MKOS-MEFs-Control cultures 20 days after the initiation of doxycycline treatment. (G): Model of direct (red) and indirect (gray) interactions between Cited2 and core pluripotency. Results in (A), (C), and (F) are presented as the mean ± SEM of three independent experiments with at least two different biological replicates. Abbreviations: AP, alkaline phosphatase; MEF, mouse embryonic fibroblast.

Having demonstrated the requirement for *Cited2* in the maintenance of ESC self-renewal, we next wanted to establish its role in the induction of the pluripotent state. To circumvent premature senescence caused by *Cited2* deficiency in primary MEFs [26], we immortalized *Cited2*^fl/fl^*;Rosa26CreER^T2^* and *Cited2*^fl/fl^ (or *Cited2*^+/+^*;Rosa26CreER^T2^*) control MEFs by serial passaging and then treated them with 4HT (Fig. 7A). To deliver *Myc*, *Klf4*, *Oct4*, and *Sox2* (MKOS factors) to MEFs we used a *piggyBac* (PB) transposon-based primary reprogramming system [27]. Briefly, *Cited2*^Δ/Δ^ and control MEFs were transfected with PB transposon carrying a doxycycline-inducible *MKOS-IRES-mOrange* cassette, a constitutively active *CAG-rtTA* transactivator construct and a transposase expression vector [27]. The expression of the MKOS factors and mOrange was activated by the addition of doxycycline to the culture medium. The frequency of mOrange positive MEFs was measured by flow cytometry at day 2 after transfection and the initiation of doxycycline treatment revealed a similar expression in *Cited2* knockout and control MEFs (Fig. 7B). We found that 15 days after the initiation of the reprogramming process the number of colonies expressing the early reprogramming marker AP was drastically reduced in *Cited2*^Δ/Δ^ cultures compared to control cultures (Fig. 7C). The expression of endogenous Nanog in colonies arising from control MEFs was verified by immunofluorescence, demonstrating efficient reprogramming of control MEFs (Fig. 7D). Finally, the results obtained in *Cited2*^Δ/Δ^ MEFs were corroborated using primary MEFs with shRNA-mediated *Cited2* knockdown (not shown). Therefore, *Cited2* is critically required for MEF reprogramming to iPS cells.

We next asked whether *Cited2* can increase the efficiency of iPS generation. For this purpose, we used secondary reprogramming system in which all MEFs harbor a doxycycline-inducible MKOS cassette and the expression of MKOS can be monitored by an mOrange reporter [27]. This system also harbors the *Nanog-eGFP* reporter that allows detecting the activation of the endogenous *Nanog* promoter. For simplicity, these MEFs will be referred to as MKOS-MEFs. To test the impact of Cited2 on reprogramming, we transduced MKOS-MEFs with MSCV retroviruses expressing *Cited2* or empty control retroviruses, and simultaneously added doxycycline to these cultures. We found that *Cited2* overexpression in MKOS-MEFs promoted an early emergence of Nanog-eGFP positive colonies in comparison to the control cells (Fig. 7E), and increased the total number of colonies expressing AP (Fig. 7F). These findings taken together imply that *Cited2* is required for reprogramming of MEFs to iPS cells, can accelerate the emergence of Nanog positive cells, and enhances the frequency of iPS cell generation.

## Discussion

Taken together, the prime effect of Cited2 depletion in E14TG2A, E14/T, and newly derived ESC lines was the impairment of self-renewal, proliferation, cell survival, and increased spontaneous differentiation. Amongst the core pluripotency genes assessed, only Nanog expression was affected as early as 48 hours after Cited2 depletion. We demonstrated that *Nanog*, *Klf4*, and *Tbx3* are direct target genes of Cited2 and Nanog constitutive expression rescued the cellular defects caused by Cited2 depletion, suggesting that these defects are, at least in part, the consequence of reduced expression of Nanog (Fig. 7G). Indeed, Nanog is a molecular gatekeeper suppressing spontaneous differentiation of ESC, which is essential for the pluripotency gene regulatory network stability and prolonged decrease of its expression secures cell fate commitment [35]. Moreover, Nanog depletion triggers stochastic early changes in ESC gene expression [35], which might account for some inconsistency observed in the expression of pluripotency related genes such as *Klf4* or *Tbx3* when we modulated Cited2 expression levels, since we demonstrate that Nanog is directly regulated by Cited2. Cited2 knockdown led to the spontaneous differentiation of E14TG2A ESC, and in addition to Nanog, a decrease of pluripotency markers such as Oct4 was observed at day 6, but not at day 2 after Cited2 depletion, suggesting that Oct4 downregulation might result from ESC differentiation rather than a direct effect of Cited2 on Oct4 expression. However, a deeper understanding of Oct4-Cited2 reciprocal regulation might be of interest since an inverse correlation of expression has been reported between these factors [28,36]. Moreover, our results also suggest that the levels of Cited2 expression influence ESC fate. Indeed, while a decrease in *Cited2* expression may favor ESC differentiation (as observed in E14TG2A ESC *Cited2* knockdown), a lack of *Cited2* expression may compromise ESC viability (as demonstrated in *Cited2* knockout ESC).

Although the immediate effect of *Cited2* deletion in ESC is the loss of their self-renewal capacity, a recent study has characterized *Cited2*-null ESC obtained by in vitro homologous recombination from a *Cited2* heterozygous ESC line [28]. We were also able to expand *Cited2*-deficient ESC that adapted to culture upon serial passaging. In contrast to the phenotypes we obtained upon acute loss of *Cited2*, *Cited2*-null ESC previously reported by Li et al. [28] showed normal self-renewal capacity in the presence of LIF and differentiation defect upon removal of LIF. This discrepancy might have come from the selection process, in which only a small number of cells that have bypassed the survival and self-renewal defects adapted to culture and expanded after deletion of *Cited2*. A comparable situation occurs when *Nanog* is depleted in ESC expressing low levels of endogenous Nanog [37]. Indeed, although Nanog-deficient ESC are overtly prone to differentiation, undifferentiated ESC lacking Nanog persist upon continuous passaging and expresses normal levels of pluripotency markers. Variations in Cited2 expression and its impact on feedback loops, ESC heterogeneity, and cell fate commitment that are Nanog-dependent [35] merit further investigations.

In this study, we have also demonstrated that *Cited2* is important for MKOS-mediated reprogramming of MEFs to iPS cells. When and how Cited2 plays pivotal roles during reprogramming remains to be identified. Given that in the primary reprogramming experiments we used immortalized *Cited2*-deficient MEFs that have bypassed senescence pathways, senescence is an unlikely explanation for their inability to undergo reprogramming. Considering that Nanog is important for efficient reprogramming [38,39], it is likely that Cited2 is involved in Nanog-dependent pathways during MKOS-mediated generation of iPS cells. Furthermore, we demonstrated that *Cited2* is required for and directly enhances the expression of *Tbx3* [40], a transcription factor that accelerates reprogramming into iPS cells. These data, taken together with our observations indicating that Cited2 accelerates and increases the efficiency of reprogramming, suggest that one function of Cited2 is to control *Nanog* and *Tbx3* expression during iPS cell generation.

## Conclusion

Collectively, we demonstrate that Cited2 is a key regulator of ESC survival and self-renewal. We also show that Cited2 is a key component of the transcriptional regulatory network emphasizing Nanog, Tbx3, and Klf4 expression by directly binding the gene loci. Our data, together with Cited2 sufficiency to maintain undifferentiated ESC [12,13], and its requirement for hematopoietic stem cell maintenance [10,11] highlight the importance of Cited2 as a master regulator of adult and ESC fates.
