# Supplementary material for: Acute Loss of Cited2 Impairs Nanog Expression and Decreases Self-Renewal of Mouse Embryonic Stem Cells
Source: Stem Cells. 2014 Nov 6;33(3):699–712. doi: 10.1002/stem.1889 (PMC4583779; doi:10.1002/stem.1889)
Supplement: Supplementary file 1 — Supporting Information [file stem0033-0699-sd1.docx]

**Acute loss of Cited2 impairs Nanog expression and decreases self-renewal of mouse embryonic stem cells**

Kamil R. Kranc^1,7^, Daniel V. Oliveira^2^, Alejandro Armesilla-Diaz^1^, Ivette Pacheco-Leyva^2^, Ana Catarina Matias^2^, Ana Luisa Escapa^2^, Chithra Subramani^1^, Helen Wheadon^3^, Marlene Trindade^2^, Jenny Nichols^4^, Keisuke Kaji^1^, Tariq Enver^5,7^and José Bragança^2,6,7,8^

**SUPPLEMENTAL MATERIAL**

**REFERENCES**

**Chen, C.-m., Bentham, J., Cosgrove, C., Braganca, J., Cuenda, A., Bamforth, S.D., Schneider, J.E., Watkins, H., Keavney, B., Davies, B., and Bhattacharya, S. 2012. Functional Significance of SRJ Domain Mutations in CITED2. *PLoS ONE* 7(10): e46256.**

**Chen, Y., Carlson, E.C., Chen, Z.-Y., Hamik, A., Jain, M.K., Dunwoodie, S.L., and Yang, Y.-C. 2009. Conditional Deletion of Cited2 Results in Defective Corneal Epithelial Morphogenesis and Maintenance. *Dev Biol* 334(1): 243-252.**

**Chen, Y., Haviernik, P., Bunting, K.D., and Yang, Y.-C. 2007. Cited2 is required for normal hematopoiesis in the murine fetal liver. *Blood* 110(8): 2889-2898.**

**Ivanova, N., Dobrin, R., Lu, R., Kotenko, I., Levorse, J., DeCoste, C., Schafer, X., Lun, Y., and Lemischka, I.R. 2006. Dissecting self-renewal in stem cells with RNA interference. *Nature* 442(7102): 533-538.**

**Ogawa, K., Saito, A., Matsui, H., Suzuki, H., Ohtsuka, S., Shimosato, D., Morishita, Y., Watabe, T., Niwa, H., and Miyazono, K. 2007. Activin-Nodal signaling is involved in propagation of mouse embryonic stem cells. *J Cell Sci* 120(1): 55-65.**

**Parisi, S., Passaro, F., Aloia, L., Manabe, I., Nagai, R., Pastore, L., and Russo, T. 2008. Klf5 is involved in self-renewal of mouse embryonic stem cells. *J Cell Sci* 121: 2629-2634.**

**Sankar, N., Baluchamy, S., Kadeppagari, R.-K., Singhal, G., Weitzman, S., and Thimmapaya, B. 2008. p300 provides a corepressor function by cooperating with YY1 and HDAC3 to repress c-Myc. *Oncogene* 27(43): 5717-5728.**

**Silva, J., Nichols, J., Theunissen, T.W., Guo, G., van Oosten, A.L., Barrandon, O., Wray, J., Yamanaka, S., Chambers, I., and Smith, A. 2009. Nanog Is the Gateway to the Pluripotent Ground State. *Cell* 138(4): 722-737.**

**Zhong, X. and Jin, Y. 2009. Critical roles of coactivator p300 in mouse embryonic stem cell differentiation and nanog expression. *J Biol Chem* 284(3): 9168-9175.**
