# Supplementary material for: Acute Loss of Cited2 Impairs Nanog Expression and Decreases Self-Renewal of Mouse Embryonic Stem Cells
Source: Stem Cells. 2014 Nov 6;33(3):699–712. doi: 10.1002/stem.1889 (PMC4583779; doi:10.1002/stem.1889)
Supplement: Supplementary file 2 — Supporting Information Table 1 [file stem0033-0699-sd2.docx]

**Table S1 – Primers used for qPCR, ChIP qPCR and genotyping**

| **qPCR** | **Forward primer** | **Reverse primer** | **Reference** |
| --- | --- | --- | --- |
| Brachyury | CTCTAATGTCCTCCCTTGTTGCC | TGCAGATTGTCTTTGGCTACTTTG | (Ivanova et al. 2006) |
| Cdx2 | CCTGCGACAAGGGCTTGTTTAG | TCCCGACTTCCCTTCACCATAC | (Ivanova et al. 2006) |
| Cited2#1 | CGCATCATCACCAGCAGCAG | CGCTCGTGGCATTCATGTTG | (Chen et al. 2007) |
| Cited2#2 | AAATCGCAAAGACGCAAGGACTGG | ATGCGGGCTCGGGAACTGC |  |
| Cited2#3 | atcgcaaagacggaagga | tgctgctggtgatgatgc | * |
| c-myc | AAGGACTATCCAGCTGCCAAGAG | TCCTTTTCAGAGGTGAGCTTGTG | (Ogawa et al. 2007) |
| Fgf5 | CTGTATGGACCCACAGGGAGTAAC | ATTAAGCTCCTGGGTCGCAAG | (Ivanova et al. 2006) |
| Foxa2 | GGCCCAGTCACGAACAAAGC | CCCAAAGTCTCCACTCAGCCTC | (Ivanova et al. 2006) |
| Gapdh | TCCCACTCTTCCACCTTCGATGC | GGGTCTGGGATGGAAATTGTGAGG | (Ivanova et al. 2006) |
| Gata6 | ACAGCCCACTTCTGTGTTCCC | GTGGGTTGGTCACGTGGTACAG | (Ivanova et al. 2006) |
| Gsc | AAACGCCGAGAAGTGGAACAAG | AAGGCAGGGTGTGTGCAAGTAG | (Ivanova et al. 2006) |
| Klf4 | CCACCAGGACTACCCCTACA | GGGGACTTGTGACTGCATCT | (Chen et al. 2009) |
| Lefty2 | TACCCACGTGAGGTCCCAGTAT | AGGTGAGTGGAGGTCTCTGACA | (Ogawa et al. 2007) |
| Nanog#1 | CTCATCAATGCCTGCAGTTTTTCA | CTCCTCAGGGCCCTTGTCAGC | (Zhong and Jin 2009) |
| Nanog#2 | AGGCTTTGGAGACAGTGAGGTGC | TACCCTCAAACTCCTGGTCCTTC | (Ivanova et al. 2006) |
| Nodal | TGGCGTACATGTTGAGCCTCT | TGAAAGTCCAGTTCTGTCCGG | (Ogawa et al. 2007) |
| Oct4 | GCAGGAGCACGAGTGGAAAGCAAC | CAAGGCCTCGAAGCGACAGATG | (Ivanova et al. 2006) |
| Rex1 | TTGGGGCGAGCTCATTACTT | TTGCCACACTCTGCACACAC | (Silva et al. 2009) |
| Sox1 | ATGCACCGCTACGACATGGG | GCTCCGACTTGACCAGAGATCC | (Ivanova et al. 2006) |
| Sox17 | AAGAAACCCTAAACACAAACAGCG | TTTGTGGGAAGTGGGATCAAGAC | (Ivanova et al. 2006) |
| Sox2 | CGAGATAAACATGGCAATCAAATG | AACGTTTGCCTTAAACAAGACCAC | (Ivanova et al. 2006) |
| TBP | ggcggtttggctaggttt | gggttatcttcacacaccatga | * |
| Tbx3 | TTATTTCCAGGTCAGGAGATGGC | GGTCGTTTGAACCAAGTCCCTC | (Ivanova et al. 2006) |

| **ChIP**  **qPCR** | **Forward primer** | **Reverse primer** | **Reference** |
| --- | --- | --- | --- |
| c-myc  PII | CGCTGCGCCCGAACAACC | CCTCAGCCCCGCAGCCCAGTA | (Sankar et al. 2008) |
| Gapdh | CAAGGCTGTGGGCAAGGT | TCACCACCTTCTTGATGTCATCA | (Chen et al. 2012) |
| Klf4 A | TGGGGAATGGGAAAAGGAGTAATA | CTGCGCTGGGAAGAGGAG |  |
| Klf4 B | CAAAATGAAGAGTGCGAGTGC | ATTTACATTGGGGGTGGGGGAGTG |  |
| Klf4 C | CGCTCTGCTCCCGTCCTTCTCCAC | CGCCCATACACCCCACCCCATCTG |  |
| Nanog  DE | GGCAAACTTTGAACTTGGGATGTGGAAATA | CTCAGCCGTCTAAGCAATGGAAGAAGAAAT | (Zhong and Jin 2009) |
| Nanog  PE | GAGGATGCCCCCTAAGCTTTCCCTCCC | CCTCCTACCCTACCCACCCCCTATTCTCCC | (Parisi et al. 2008) |
| Nanog  “Stat3” | GAGGACTCGCATGCATTTTGTTTCTA | AAGGGCGACGTAATTTTGGTAAGC | (Zhong and Jin 2009) |
| Oct4  CR1 | AGCAACTGGTTTGTGAGGTGTCCGGTGAC | CTCCCCAATCCCACCCTCTAGCCTTGAC | (Parisi et al. 2008) |
| Oct4  CR4 | GGAACTGGGTGTGGGGAGGTTGTA | AGCAGATTAAGGAAGGGCTAGGACGAGAG | (Parisi et al. 2008) |
| Tbx3 A | TGGGGGCTCGGTCTCAGTATGTCG | AATCTTTCTGCCCGCTGCCTGCTC |  |
| Tbx3 B | CCTCGCTCCCTCCCCCTTCCTC | GCTCGCCGCGGCTCCTCAG |  |
| Tbx3 C | TCCAGAGCCGCCGAGCAGAC | GTAGGCAAGTAGAGGGAGGGAGAG |  |

| **Genotyping** | **Forward primer** | **Reverse primer** |
| --- | --- | --- |
| Cited2 fl set#1 | GTCTCAGCGTCTGCTCGTTT | CTGCTGCTGTTGGTGATGAT |
| Cited2 fl set#2 | GTCTCAGCGTCTGCTCGTTT | CGCTCGTGGCATTCATGTTG |
| Cited2 Δ | TCTCTCGGGGGTGGTGGTC | CCTCCGACCTGCAGGAATTAG |

*Primers and probes, designed online with the Universal Probe Library (www.roche-applied-science.com/sis/rtpcr/upl; Roche Applied Science)
