# Supplementary material for: Acute Loss of Cited2 Impairs Nanog Expression and Decreases Self-Renewal of Mouse Embryonic Stem Cells
Source: Stem Cells. 2014 Nov 6;33(3):699–712. doi: 10.1002/stem.1889 (PMC4583779; doi:10.1002/stem.1889)
Supplement: Supplementary file 3 — Supporting Information Figure 1 [file stem0033-0699-sd3.doc]

**SUPPLEMENTAL FIGURE LEGENDS**

**Figure S1. C2^fl/fl^ ESC have pluripotent features**

(A) C2^fl/fl^ ESC were differentiated by hanging drop method and removal of LIF. Expression of pluripotency transcripts (black bars) and differentiation markers (white bars) were determined by qPCR in C2^fl/fl^ ESC treated with 1uM 4-hydroxytamoxifen (4HT) or ethanol (vehicle for 4HT) for 48 hrs. Expression levels are normalized for Gapdh and reported as a fold of expression relative to C2^fl/fl^ ESC treated with ethanol. Results are presented as the mean ± s.e.m. of three independent biological replicates (each performed in technical duplicate).The panel shows that markers of pluripotency decreased while markers of differentiation are expressed. (B) The emergence of cardiac beating foci from C2^fl/fl^ ESC-derived embryoid in culture is presented at the indicated days. (C) Expression of pluripotent gene transcripts determined by qPCR in C2^fl/fl^, and C2^fl/fl^ ESC stably transfected with a Cre-ERt expressing plasmid, the ESC lines C2^fl/fl^[Cre]A, C2^fl/fl^[Cre]B and C2^fl/fl^[Cre]C, treated with 1uM 4HT or ethanol (vehicle for 4HT) for 48hrs. Expression levels are normalized for *Gapdh* and reported to relative to the expression in C2^fl/fl^ ESC treated with ethanol which is set at 1. Data are the mean ± s.e.m. of three independent biological replicates (each performed in technical duplicate). (D) Proliferation of E14TG2A cells stably transfected with a plasmid expressing Cre-ERt (E14TG2A[Cre]) after 0.5uM 4HT or ethanol (vehicle) treatment, and E14TG2A cells transiently transfected with a plasmid expressing Cre and GFP (E14TG2A/Cre) compared to the control cells (E14TG2A/Control). GFP postive cells were isolated by FACS. Results are presented as cumulative population doublings (CPD) of cells plated at 10000 cells per gelatinized wells (6-well plate) at day 0. Results are shown as the mean ± s.e.m. of three independent biological replicates (each performed in technical duplicate). (E) Proliferation of C2^fl/fl^ ESC cells transiently transfected with a plasmid expressing Cre and GFP (C2^Δ/Δ^/CreErt) compared to the control cells expressing GFP without Cre. GFP postive cells were isolated by FACS. Results are presented as the mean ± s.e.m. of two independent biological replicates performed in technical duplicates. (F) CPD of cells obtained as described in Figure 2C cultured in medium supplemented with LIF, but with FBS from another supplier. (G) Representative morphology and Alkaline Phosphatase (AP) activity of C2^fl/fl^[Cre]/Control, C2^Δ/Δ^[Cre]/Control, C2^fl/fl^[Cre]/CITED2 and C2^Δ/Δ^[Cre]/CITED2 ESC 6 days after ethanol or 4HT treatment. (H) Proliferation of E14TG2A cells (right panel) was determined after transfection of a plasmid expressing a shRNA specific for Cited2 which induces the decrease of Cited2 expression by more than 2 fold after 2 days as shown by qPCR (left panel). Data are the mean ± s.e.m. of two independent experiments performed in technical duplicates.

**Figure S2. A fraction of mouse ESC survive acute loss of Cited2**

(A) Schematic representation of the *Cited2* locus in C2^fl/fl^ and C2^Δ/Δ^ ESC and localization of primers used for genotyping. (B) Results of PCR genotyping of 6 independent C2^Δ/Δ^ ESC clones. C2^fl/fl^ ESC were transiently transfected with a Cre-expressing plasmid and subsequently replated and grown in culture for ~2 weeks before being isolated by FACS based on the β-Galactosidase expression levels using Fluorescein di-β-DGalactopyranoside (FDG). (C) Three ESC C2^Δ/Δ^ [HD2], C2^Δ/Δ^[MG5] and C2^Δ/Δ^ [LA11] presenting respectively high, medium and low levels of β-galactosidase activity at the time of isolation were grown in the presence or the absence of LIF for 4 days. The morphology of the parental control cell line C2^fl/fl^ ESC is also presented. (D) Alkaline phosphatase staining in C2^fl/fl^, C2^Δ/Δ^ [HD2], C2^Δ/Δ^[MG5] and C2^Δ/Δ^ [LA11] grown in the presence or the absence of LIF for 4 days. (E) Cumulative number of C2^fl/fl^, C2^Δ/Δ^[HD2], C2^Δ/Δ^[MG5] and C2^Δ/Δ^ [LA11] cells grown at the indicated time points in the presence or the absence of LIF. (F) Fraction of C2^fl/fl^, C2^Δ/Δ^ [HD2], C2^Δ/Δ^[MG5] and C2^Δ/Δ^ [LA11] stem cell colonies formed when grown in the presence or the absence of LIF. (G) Detection of the Cited2 protein in C2^fl/fl^ parental cells and and absence in C2^Δ/Δ^ [HD2], C2^Δ/Δ^[MG5] and C2^Δ/Δ^[LA11] stem cells by western blotting. (H) mRNA was isolated and qPCR was performed to determine the level of the indicated gene transcripts in C2^fl/fl^, C2^Δ/Δ^ [HD2], C2^Δ/Δ^[MG5] and C2^Δ/Δ^  [LA11] stem cells.Expression level of each indicated gene was normalized for *Gapdh* and referred to their expression in C2^fl/fl^ cells which is set at 1. Error bars indicate mean ± s.e.m. of at least three biological replicates.
